# Serum amyloid A (SAA) and Interleukin-6 (IL-6) as the potential biomarkers for gastric cancer

**DOI:** 10.1097/MD.0000000000031514

**Published:** 2022-10-28

**Authors:** Yongwang Hou, Weidong Zhao, Zhicong Yang, Bin Zhang

**Affiliations:** a Clinical Laboratory, The First Affiliated Hospital of Hebei North University, Zhangjiakou City, Hebei Province, China; b Beijing Aerospace General Hospital, Beijing, China; c Central Laboratory, The First Affiliated Hospital of Hebei North University, Zhangjiakou City, Hebei Province, China.

**Keywords:** biomarkers, gastric cancer, Interleukin-6, serum amyloid A

## Abstract

To explore serum amyloid A (SAA) and interleukin-6 (IL-6) as potential diagnostic biomarkers for gastric cancer (GCa) and the application value of the combined diagnosis of SAA, IL6, and Cancer embryonic antigen. Serum samples were collected before the initial surgery from 159 patients comprising samples from 122 patients with GCa and 37 patients with benign gastric disease. All patients were hospitalized at Beijing Aerospace General Hospital in China between 2018 and 2020. The IL-6 and SAA levels were assessed using standard laboratory protocols. The levels of SAA and IL-6 were significantly higher in patients with GCa than in controls. Compared with the healthy group, the concentration of SAA and IL-6 in FIGO III–IV group were significantly higher and the difference were statistically significant. In addition, significant differences were observed between the FIGO III–IV group and FIGO I–II groups. The Receiver operating characteristic (ROC) curve for the combined detection of SAA, IL-6, and Cancer embryonic antigen showed an area under the curve (AUC) of 0.948, sensitivity of 91.0%, and specificity of 89.2%. Spearman’s correlation analysis indicated obvious correlations among the levels of serum SAA, IL-6, advanced FIGO stage, lymphatic invasion, and distant metastasis. AA and IL-6 may serve as useful biomarkers for poor prognosis of GCa. Clinical diagnosis combined with SAA and IL-6 may help assess therapeutic outcomes.

## 1. Introduction

Gastric cancer (GCa) is a common malignant tumor of the digestive system with high mortality, poor prognosis, and complex inducements. In China, GCa is the second most commonly diagnosed cancer, with an incidence of 15.8% and an estimated 0.68 million new GCa cases in 2015.^[1]^ Several factors are suspected to play a role in the development of GCa, including the effects of diet, intake of smoked, exogenous chemicals, genetic factors, and infectious agents.^[2]^ Early diagnosis and treatment are helpful in obtaining the best prognosis for patients with GCa. Tumor markers are biomolecules crucial for early tumor screening. Previous studies have found that lncRNA XIST could be a new biomarker for GCa, which promotes proliferation, migration, and invasion of GCa cells by targeting miR-337.^[3]^ Clinical practice has shown that cancer embryonic antigen (CEA) has the highest expression rate in gastrointestinal tumors. However, when the above tumor marker is used alone to screen for gastrointestinal tumors, the sensitivity and specificity of each indicator are poor and the tumor prediction effect is insufficient. Therefore, it is of great importance to identify new biomarkers in the serum, which can help in diagnosing and predicting GCa. Clinical and epidemiological studies have shown a link between GCa and chronic inflammation; thus, the pathogenesis of GCa represents inflammation-driven malignancy.^[4]^ In recent years, chronic inflammation has been confirmed to be associated with tumor progression, and many inflammatory factors can be used as diagnostic and prognostic markers for specific tumors.^[5]^ Studies have shown that inflammation can be a chronic process that promotes angiogenesis and cell proliferation. Therefore, inflammation may play a clear role in carcinogenesis and pathogenesis.

Serum amyloid A (SAA), an acute phase protein, is mainly synthesized in the liver and dramatically increases during inflammatory diseases.^[6]^ Serum SAA levels can be elevated more than 1000 folds during inflammation.^[7]^ Therefore, SAA has long been regarded as a sensitive marker of inflammation.^[8]^ Convincing evidence has shown that chronic infection and inflammation, especially the biosynthesis and secretion of proinflammatory cytokines, are associated with cancer.^[9]^ Moreover, it is reported that the concentration of SAA is significantly high in different types of cancer including lung cancer,^[10]^ breast cancer,^[11]^ uterine cervical cancer,^[12]^ ovarian cancer,^[13]^ renal cancer,^[14]^ and others.^[15,16]^

Interleukin-6 (IL-6) is a soluble mediator with a pleiotropic effect on inflammation, the immune response, and hematopoiesis. After IL-6 is synthesized in a local lesion in the initial stage of inflammation, it moves to the liver through the bloodstream, followed by the rapid induction of an extensive range of acute phase proteins, such as C-reactive protein, SAA, fibrinogen, haptoglobin, and a1-antichymotrypsin.^[17]^ IL-6 is promptly and transiently produced in response to infections and tissue injuries, and contributes to host defense through the stimulation of acute phase responses, hematopoiesis, and immune reactions. Recent studies have suggested that IL-6 is involved in the genesis and development of tumors. Measurement of serum levels of IL-6 in patients with pancreatic cancer revealed that it is more highly expressed in these patients than in healthy controls.^[18]^ Recent data from the literature revealed the critical role of IL-6 and its signaling pathways in pancreatic cancer oncogenesis through JAK2-STAT3 activation.^[19]^ Furthermore, IL-6 participates in pancreatic cancer oncogenesis, metastasis, treatment resistance via mesothelin-protein kinase B-NF-kB (MSLN-Akt-NF-kB), and overexpression of IL-6-Induced myeloid leukemia cell differentiation protein (IL-6-Mcl-1).^[20]^

In this study, we measured the concentrations of SAA and IL-6 and assessed the relationship between SAA and IL-6 and the prognosis of GCa patients. Furthermore, we demonstrated the advantages of SAA and IL-6 in diagnosing GCa combinations of CEA, and found that SAA and IL-6 could be potential biomarkers for GCa.

## 2. Material and methods

### 2.1. Patients and samples

Ethical approval for the study was obtained from the medical ethics committee of The First Affiliated Hospital of Hebei North University (K2021139). All participants have informed consent. Blood samples were collected using standardized procedures. After obtaining patients’ approval, a total of 159 serum samples were collected before the initial surgery, including 122 patients (average age 53.67 ± 1.23 years) with GCa and 37 patients (average age 52.08 ± 2.42 years) with gastric benign disease. All data were obtained from Beijing Aerospace General Hospital in China between 2018 and 2020. Patients with rheumatoid arthritis and acute inflammatory infections were excluded from this study. Thirty healthy age-matched individuals (average age 51.23 ± 2.86 years) were selected as normal controls. The results of the 1-way ANOVA showed that there was no statistically significant difference among the 3 groups of subjects (*P* = .395). Venous blood samples were collected in pyrogen-free tubes, allowed to clot at 4°C for 1 hour, and centrifuged at 2000 × g for 10 minutes. The upper serum layers were carefully obtained, divided into separate vials, and stored at −20°Cuntil the assay was conducted.

### 2.2. The Assay of SAA and IL-6

were detected using a commercial kit for serum amyloid A protein assay (Ningbo Purebio Biotechnology Co, Ltd, Ningbo, China) and Automatic Analyzer H7180ID, following the manufacturer’s instructions. The serum level of IL-6 was determined using a commercial kit for interleukin 6 assay (Ningbo Purebio Biotechnology Co, Ltd) and the Caris200 System, following the manufacturer’s instructions. The instruction states that if the concentration of the sample exceeds the linear range, it should be diluted with normal saline and remeasured.

### 2.3. Statistical analysis

Mann–Whitney *U* test was used to compare the differences between 2 groups, and Kruskal–Wallis test was used to compare the differences between more than 2 groups. Correlation coefficients were determined using Spearman analyses. Logistic regression was used to test the univariate and multivariate analyses of clinicopathological factors related to SAA and IL6. Statistical significance was set at *P* < .05. All statistical analyses were performed using IBM SPSS 22.0 (IBM Corp, Armonk, NY) and GraphPad Prism 7 (San Diego, CA).

## 3. Results

### 3.1. The Concentration of serum SAA and IL-6 in GCa

**Figure 1.**
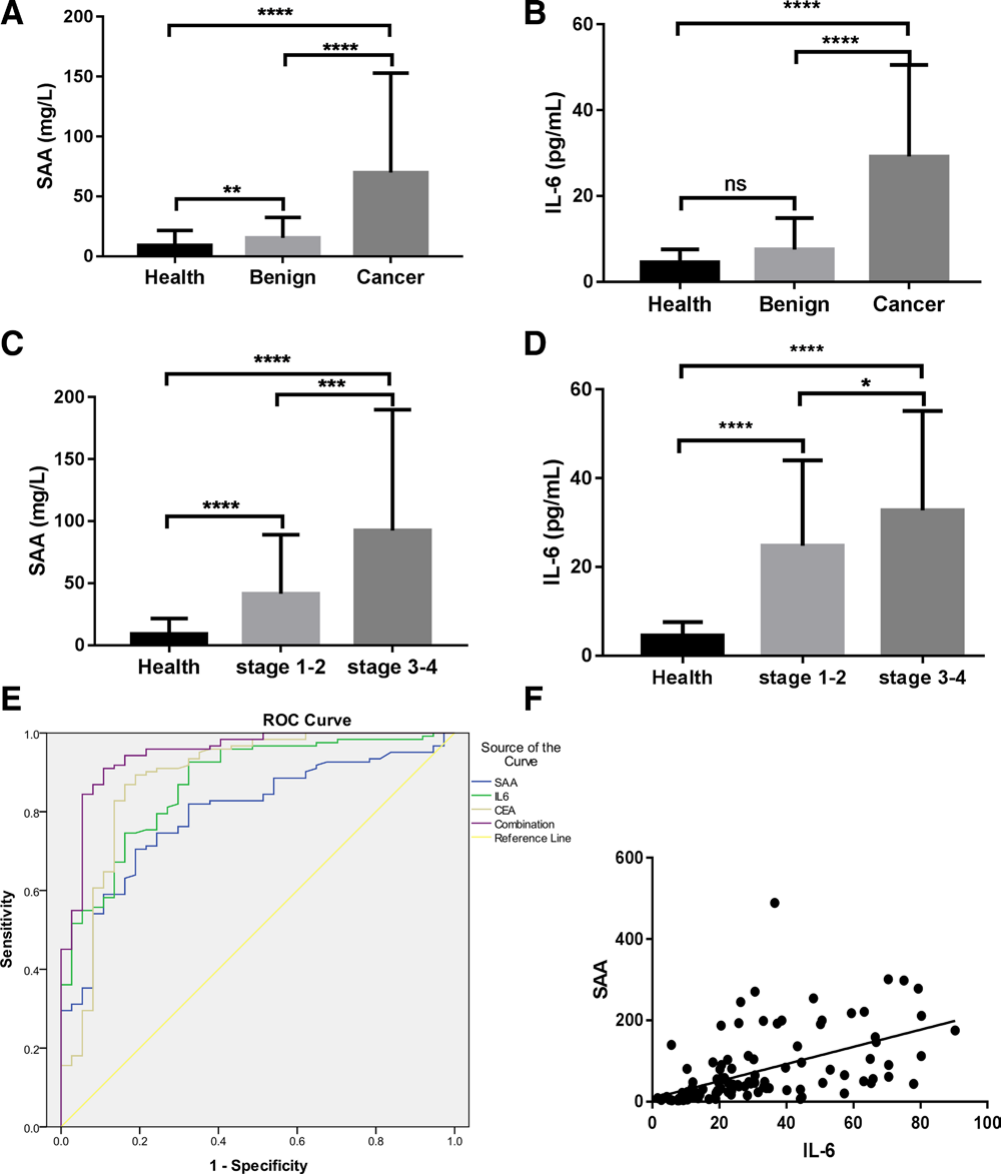
SAA and IL-6 as prognostic biomarker in GCa. (A, B) The concentrations of SAA and IL-6 in health, benign and GCa. (C, D) The concentrations of SAA and IL-6 in health, FIGO I–II group And FIGO III–IV group. ****, *P* < .0001, **, *P* < .01, *, *P* < .05, ns, *P* > .05. (E) ROC curve of sensitivity versus specificity of SAA, IL-6, CEA and combinations of 3 makers. (F) A positive correlation between serum IL-6 and SAA, *R*^2^ = 0.295, *P* < .0001. CEA = Cancer embryonic antigen, GCa = gastric cancer, IL-6 = Interleukin-6, SAA = serum amyloid A.

To confirm that the expression levels of SAA and IL-6 are improved in GCa, the concentrations of SAA, IL-6, and CEA in healthy, benign gastric disease, and GCa tissues were examined by chemiluminescent immunoassay, as shown in Table 1. Compared with the healthy group, the concentration of SAA and IL-6 in the GCa group were significantly higher and the difference were statistically significant (*P* = .000, *P* = .000). In addition, there was a significant difference between the GCa and benign gastric disease groups (*P* = .000, *P* = .000) (Fig. 1A and B). Significant associations were observed between advanced FIGO stage and SAA and IL-6 levels. Compared with the healthy group, the concentration of SAA and IL-6 in FIGO III–IV group were significantly higher and the difference were statistically significant (*P* = .000, *P* = .000). In addition, the FIGO I–II group was different from the healthy group (*P* = .000, *P* = .000). In addition, there was a significant difference between the FIGO III–IV group and FIGO I–II groups (*P* = .000, *P* = .033) (Fig. 1C and D).

**Table 1:** The median serum SAA, IL-6 and CEA concentration.

Group	N	SAA [mg/L, M (QR)]	IL6 [pg/mL, M (QR)]	CEA [ng/mL, M (QR)]
Health	30	5.75 (3.70, 8.95)	3.67 (1.84, 7.39)	4.61 (2.90, 7.00)
Benign	37	9.30 (5.75, 18.30)	3.87 (2.43, 11.15)	7.90 (3.71, 11.77)
Gastric Cancer	122	37.50 (15.28, 90.15)	23.57 (11.71, 39.86)	31.50 (20.15, 58.69)

CEA = Cancer embryonic antigen, IL-6 = Interleukin-6, SAA = serum amyloid A.

### 3.2. Serum SAA and IL-6 as the prognostic biomarker in GCa

To estimate the diagnostic power of serum SAA and IL-6 levels in GCa, we used the ROC curve for both. The ROC curves for SAA, IL-6, and CEA are shown (Fig. 1E). As shown in Table 2, the ROC curve of SAA concentration in GCa was plotted to obtain an AUC = 0.796, the cutoff value was 20.90 mg/L, and the sensitivity and specificity were 70.5% and 81.1%, respectively. The ROC curve for the diagnosis of GCa based on IL-6 concentration was plotted. The AUC was 0.871; cutoff value was 11.91 pg/L; the sensitivity was 74.6%, and 83.8%, respectively. The ROC curve of CEA for the diagnosis of GCa showed an AUC of 0.886, cutoff value at this time was 15.66 ng/mL, sensitivity of 86.9%, and specificity of 83.8%. In the combined detection of SAA, IL-6, and CEA, the AUC was 0.948, sensitivity was 91.0%, and specificity was 89.2%. These data suggest that SAA and IL-6 can be used as potential biomarkers, and the combined detection of SAA, IL-6, and CEA is valuable for the diagnosis of GCa. Interestingly, we observed a positive correlation between serum IL-6 and SAA (Fig. 1F). These results may provide a new direction for future research.

**Table 2:** ROC curve of the diagnostic power of serum SAA and IL-6 level for gastric cancer.

	Cutoff	AUC	YOUDEN	Sensitivity	Specificity	95% CI	
						Lower	Upper
SAA	20.90	0.796	0.516	0.705	0.811	0.721	0.871
IL-6	11.91	0.871	0.584	0.746	0.838	0.809	0.934
CEA	15.66	0.886	0.707	0.869	0.838	0.812	0.96
Combination	–	0.948	0.802	0.910	0.892	0.908	0.989

AUC = area under the curve, CEA = Cancer embryonic antigen, CI = confidence interval, IL-6 = Interleukin-6, SAA = serum amyloid A, ROC = receiver operating characteristic.

### 3.3. Serum SAA and IL-6 levels in patients with GCa after treatment

**Figure 2.**
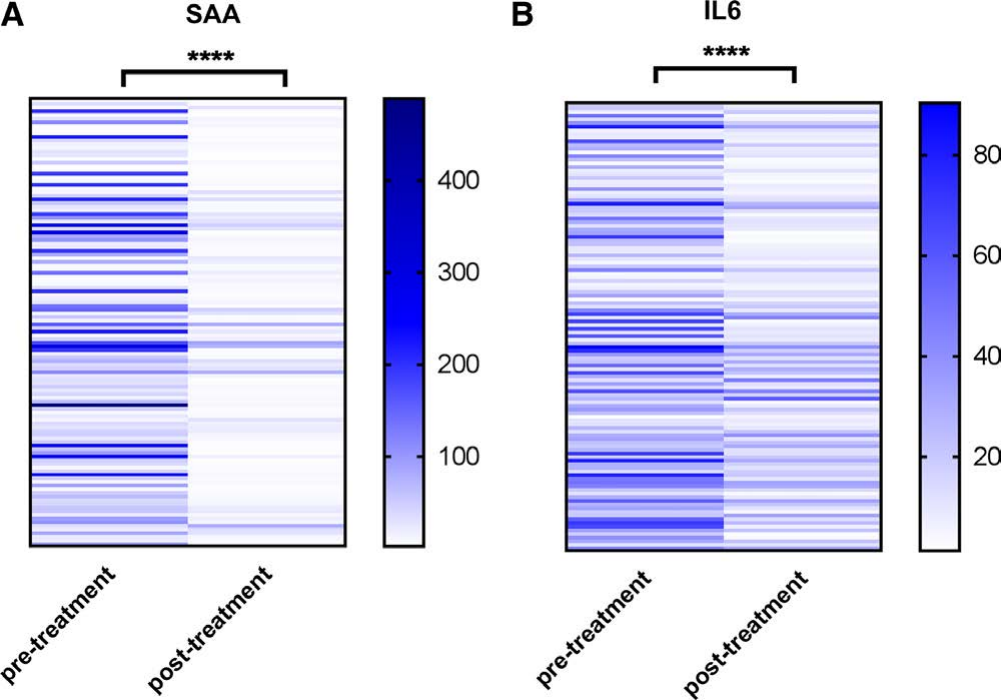
The prognostic value of SAA and IL6 in GCa. (A, B) Heat-map of SAA and IL-6 between pretreatment and post-treatment. N = 122, ****, *P* < .0001. GCa = gastric cancer, IL-6 = Interleukin-6, SAA = serum amyloid A.

To evaluate the monitoring value of SAA and IL6 in the treatment of GCa, we compared changes in serum SAA and IL6 concentrations before and after treatment. Strikingly, compared with pretreatment, the levels of serum SAA and IL-6 decreased significantly after treatment (Fig. 2A and B). Clinically, the results showed that the effect of treatment on GCa patients can be assessed by detecting the levels of serum SAA and IL-6.

### 3.4. Overexpression of serum SAA and IL-6 are associated with advanced clinical features in GCa

In total, 122 patients were included in the analysis. Details of the patients’ information are shown in Table 3, and the relationship between SAA, IL-6, and the clinicopathological characteristics of GCa were further analyzed. SAA and IL-6 expression levels were not significantly associated with age. Spearman’s correlation analysis revealed that there were significant correlations among the levels of serum SAA, IL-6, advanced FIGO stage, lymphatic invasion, and distant metastasis (Tables 3 and 4).

**Table 3:** Univariate analysis for the association of various clinicopathologica features with SAA and IL-6 expressions of patients with gastric cancer.

Feature	N	SAA [mg/L, M (QR)]	Z	*P*	IL6 [pg/mL, M (QR)]	Z	*P*
Age (years)							
<60	74	41.10 (16.73, 98.48)	−0.574	.566	23.14 (11.54, 36.74)	0.776	.438
≥60	48	37.30 (13.25, 74.13)			26.12 (11.81, 52.54)		
FIGO Stage							
I–II	54	27.50 (10.80,47.78)	−3.541	.000	19.88 (10.21, 33.27)	2.013	.044
III–IV	68	50.15 (23.93,147.33)			26.12 (14.43, 47.12)		
Lymphatic invasion							
NO	54	21.85 (8.60,42.50)	−5.059	.000	19.70 (9.51, 30.47)	2.961	.003
YES	68	58.40 (27.35,184.27)			28.49 (15.54, 55.72)		
Distant invasion							
NO	100	31.75 (11.83,56.33)	−4.801	.000	28.49 (15.54, 55.72)	3.306	.001
YES	22	151.65 (55.7218.50)			36.93 (24.40, 65.26)		

IL-6 = Interleukin-6, SAA = serum amyloid A.

**Table 4:** Spearman’s correlation analysis for the association of clinicopathologica features with serum SAA and IL-6 expressions of patients with gastric cancer.

	SAA		IL-6	
Feature	*r*	*P*	*r*	*P*
Age	0.054	.556	0.071	.440
FIGO Stage	0.466	.000	0.305	.001
Lymphatic invasion	0.436	.000	0.269	.003
Distant invasion	0.483	.000	0.305	.001

IL-6 = Interleukin-6, SAA = serum amyloid A.

## 4. Discussion

Inflammation is closely related to the occurrence and progression of tumors. During tumor development, activated inflammatory cells act as donors of reactive oxygen species and reactive nitrogen mediators, resulting in DNA damage and genomic instability.^[21]^ In return, DNA damage leads to an inflammatory response that promotes tumor progression.^[22]^ SAA, as an acute-phase protein, activates its pathway and binds to plasma high-density lipoprotein in the presence of inflammation or tumor factors, playing a key role in the adhesion, invasion, and metastasis of tumor cells.^[23]^ IL-6 is a pleiotropic inflammatory cytokine that plays conflicting roles in tumor cells. It may promote the killing of cancer cells by stimulating the antitumor activity of macrophages and preventing the apoptosis of neutrophils.^[24]^ Therefore, we measured serum SAA and IL6 in patients with GCa. We observed that compared with the healthy group, the concentrations of SAA and IL-6 in the GCa group were significantly higher, and the difference was statistically significant. These results indicate that SSA and IL-6 are highly expressed in GCa. Our findings were in agreement with a report by other authors,^[25,26]^ who showed significantly higher concentrations of IL-6 and SAA in patients with GCa than in healthy subjects.

In the present study, the levels of IL-6 and SAA increased with tumor stage. These findings are in line with a previous study,^[27]^ which showed that serum levels of IL-6 were significantly related to the stage of GCa. Another study indicated a significant association between serum SAA concentration and TNM stage.^[28]^ In addition, in the current study, the serum concentrations of SAA and IL-6 increased with distant invasion. In addition, serum levels of both proteins tested varied according to nodal metastases, and the SAA and IL-6 concentrations were correlated with the presence of lymph node metastasis. Correlation analysis revealed that there was a significant correlation between the levels of serum SAA, IL-6, advanced FIGO stage, lymphatic invasion, and distant metastasis. The study of Zhou Jielin found that the concentrations of SAA increased with the severity of cancer stages. This conclusion is consistent with ours. However, the report has shown high circulating SAA levels were markedly associated with the developing risks of cancer, especially the subject age more than 50 in GCas.^[29]^ In present study, Serum SAA concentration was not related to age of GCa patients. Our results revealed significant differences in lymph node metastasis and serum concentrations of IL-6 as well as SAA. These findings may be explained by the following mechanisms: O’Hara R, and Tamamoto T et al had reported that SAA was involved in adhesion, migration, and tissue infiltration of inflammatory cells and induced matrix metalloproteinases which could interact with degrading extracellular matrix controlling the diffusion and migration of cells.^[30–32]^ Possible mechanisms of SAA in stimulating MMP-9 might involve formyl peptide receptor like-1-mediated signaling.^[33]^ Some studies have shown that SAA can influence carcinogenesis by activating the transcription factor and nuclear factor kappa-B.^[34,35]^ SAA may also favor tumor development by limiting immune antitumor activity by stimulating the growth of regulatory T cells in a process involving IL-1*β* and IL-6 induction in monocyte.^[36]^ In contrast, IL-6 released from leukocytes was able to activate the production of IL-6 by tumor cells with the IL-6 receptor, and stimulated stromal cells promoted the secretion of molecules such as VEGF.^[37]^ It has been proven that when concentrations of IL-6 increase, SAA levels also increase and are generally associated with IL-6 and indirectly with cancer progression.^[17]^ Our data demonstrate a positive correlation between serum IL-6 and SAA levels.

The conclusion of previously study was serum levels of SAA could be of great value for early diagnosis of gastric carcinoma. The results had shown SAA at cutoff of 18.5 mg/L had the best validity to differentiate gastritis from gastric carcinoma, with AUC, sensitivity, specificity, negative predictive value, and positive predictive value of 0.99, 98%, 100%, 100%, and 98%, respectively, and higher serum levels of both SAA reflected higher tumor grade and advanced tumor stage.^[26]^ In the present study, diagnostic criteria, such as diagnostic sensitivity and specificity, as well as ROC curves for all proteins tested, were evaluated. The results indicated that the IL-6 area under the ROC curve was higher than the AUC for SAA, but slightly lower than that for CEA. In our study, the sensitivity and specificity of CEA levels were 86.9% and 83.8%, respectively. However, in the combined detection of SAA, IL-6, and CEA, the AUC was 0.948, sensitivity was 91%, and specificity was 89.2%. These data suggest that SAA and IL6 can be used as potential biomarkers, and the combined detection of SAA, IL-6, and CEA showed a good value for the diagnosis of GCa, which has not been reported.

In clinical practice, it is important to predict tumor progression after treatment. An ideal prognostic marker can lay the foundation for evaluating the clinical outcomes. The best treatment strategy for patients can be selected to avoid overtreatment or undertreatment in the clinical setting. In-Hye Ham has provided plausible evidence for crosstalk between GC cells and CAFs, wherein IL-6 is a key contributor to chemoresistance. These findings suggest the potential therapeutic application of IL-6 inhibitors to enhance the responsiveness to chemotherapy in GC.^[27]^ In this study, we observed that the serum SAA and IL-6 levels decreased significantly after treatment. These data suggest that SAA and IL-6 could be ideal predictive biomarkers for treatment outcomes and reasonable therapeutic targets for GCa.

## 5. Conclusions

Based on the current findings, we presume that SAA and IL-6 are involved in neoplastic progression and their value SAA and IL-6 in GCa. In conclusion, SAA and IL-6 may serve as useful biomarkers of poor prognosis. Clinical diagnosis combining SAA and IL-6 may help to evaluate therapeutic outcomes.

## Acknowledgments

We thank The First Affiliated Hospital of Hebei North University and the Beijing Aerospace General Hospital for their approval of the study.

## Authors’ contributions

**Conceptualization:** Yongwang Hou.

**Data curation**: Yongwang Hou.

**Data analysis:** Zhicong Yang.

**Methodology:** Weidong Zhao.

**Writing – original draft:** Yongwang Hou.

**Writing – review & editing:** Yongwang Hou, Bin Zhan.
